# Medically Managed Lyme Periprosthetic Joint Infection: A Case Report

**DOI:** 10.7759/cureus.56457

**Published:** 2024-03-19

**Authors:** Allen Saar, Shan Fairbanks

**Affiliations:** 1 Family Medicine, Edward Via College of Osteopathic Medicine, Christiansburg, USA; 2 Family Medicine, Carilion Clinic, Dublin, USA

**Keywords:** pji, total knee arthroplasty, tka, periprosthetic joint infection, lyme arthritis

## Abstract

Arthritis associated with Lyme disease is frequent in regions of the United States where the illness is widespread; nonetheless, periprosthetic joint infections (PJI) caused by Lyme are exceptionally rare. As of October 2023, only five cases of Lyme PJI have been documented in the literature. Four of these cases were managed successfully with surgical intervention, while one was managed successfully with oral and IV antibiotics. Because of limited documented cases, there are no recommended treatment guidelines. This has left physicians trying to blend the treatment guidelines for Lyme arthritis, which is medically managed with antibiotics, and PJIs, which involve invasive surgical procedures. The patient in this case presented with a common presentation of acute Lyme arthritis, but it was complicated by a previous total knee arthroplasty. Treatment of this patient included three months of doxycycline 100mg BID, which resulted in complete resolution of symptoms.

## Introduction

In the northeastern region of the United States, Lyme arthritis is a common complication of late-stage Lyme disease, a tick-borne illness caused by *Borrelia burgdorferi*. The most common presentation of Lyme arthritis is intermittent episodes of swelling of larger joints, usually the knee, without constitutional symptoms [1]. According to the Infectious Diseases Society of America, the initial treatment guidelines for Lyme arthritis specify the use of doxycycline 100mg BID or amoxicillin 500mg TID [2]. While Lyme arthritis of native joints is very common in endemic areas, Lyme periprosthetic joint infections (PJIs) are surprisingly rare. PJIs are important, yet challenging, to recognize as they place tremendous strain on the patient and can substantially increase medical costs. Current recommendations for PJIs vary according to the severity and duration of the infection. Acute PJIs (<4 weeks from surgery or <3 weeks of symptoms) are treated with debridement and retention of the prosthesis. In contrast, chronic PJIs usually result in complete removal and exchange of prosthesis, often in multiple stages. These surgical interventions are accompanied by prolonged antibiotic therapy [3]. Prior to 2022, only five cases of Lyme PJI had been reported in the literature, leaving physicians with limited resources to guide the treatment of Lyme PJI patients. Of these five cases, four patients were successfully treated by surgical intervention and antibiotics, while 1 was successfully treated with intravenous (IV) and oral antibiotics only [4-8]. With this case report, we hope to add to the current literature and the discussion of Lyme PJI treatment.

## Case presentation

A 68-year-old male from southwest Virginia, with a past surgical history of left total knee arthroplasty (TKA) for degenerative joint disease 12 years prior, presented to a family medicine clinic. The patient has a past medical history of benign essential hypertension, mixed dyslipidemia, gastroesophageal reflux disease without esophagitis, paroxysmal atrial fibrillation, and benign prostatic hyperplasia (BPH) with an elevated prostate-specific antigen (PSA). The patient reported five days of suprapatellar swelling, pain, heat, and mild erythema of the left knee. He noted pain with passive flexion and extension of the knee, as well as a noticeable limp. He denied fever, chills, night sweats, and unintentional weight loss. He denied any open wounds or recent trauma. He is physically active and reported no prior problems with his left TKA.

On physical examination, limited active and passive range of motion (ROM) in flexion and extension was found, with approximately 30 degrees of flexion prior to pain and 15 degrees of extension. The left knee was positive for erythema, left suprapatellar effusion, and no point tenderness over the patella or patellar tendon. The exam was positive for point tenderness throughout, secondary to tautness, and positive for posterior fullness. Patellar grind, varus, and valgus stressing at 0 and 30 degrees, Lachman, and McMurray testing were all negative. The patient had a limping antalgic gait. Outside of an elevated blood pressure of 178/101, vitals were within normal limits. No assistive device was being used to help with ambulation.

**Figure 1:**
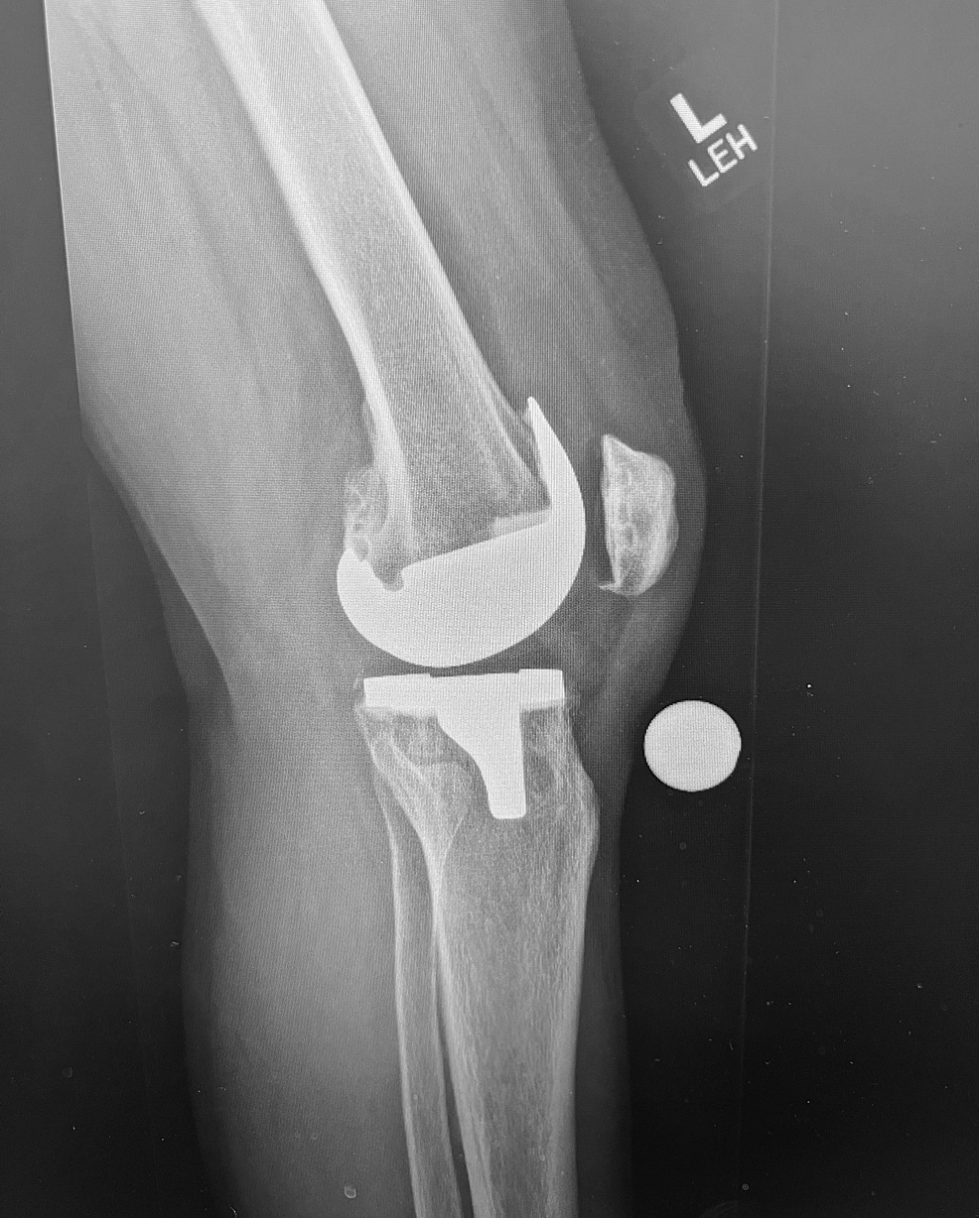
Left lateral knee x-ray demonstrating total knee arthroplasty with mild joint effusion five days post initial joint aspiration.

Laboratory data included a serum white blood cell count of 6.4 x 1,000/mL, 65.5% neutrophils, erythrocyte sedimentation rate (ESR) of 8 mm/hr (normal 0-20), and c-reactive protein (CRP) of 6.10 mg/dL (normal <1.0). An ultrasound-guided left knee aspiration was completed during the initial visit, yielding 65cc of synovial fluid. Synovial fluid was yellow and turbid with a WBC count of 14,760 cells/mm^3^, which consisted of 90% neutrophils. No crystals were seen. Culture revealed no growth after three days and the gram stain showed no organisms. Urine analysis was within normal limits. *Borrelia spp*. DNA was detected on a qualitative PCR test. Patient underwent x-ray imaging five days following aspiration, with results shown in Figure 1.

Twelve days after the initial presentation, the patient was seen by orthopedics. 23cc of synovial fluid was removed from the left knee. Repeat cultures, crystal exam, and gram stain were negative. Synovial fluid differential revealed a WBC count of 1,733 cells/mm^3^ with 1% neutrophils, 8% mononuclear cells, and 91% lymphocytes. *Borrelia spp*. DNA was detected again on a repeat qualitative PCR test.

The patient was started on doxycycline 100mg twice daily (BID) for 30 days. After completion of his 30-day antibiotic regimen, no effusion was noted upon physical exam, but the patient did have signs of pes anserinus bursitis. He was encouraged to increase activity and continue doxycycline 100mg BID for another 30 days. Upon returning to the clinic after a cumulative 60 days of doxycycline treatment, the patient reported trace nighttime left knee swelling that resolved by morning, with no effusion noted on physical examination. At this time, he was instructed to continue doxycycline for 30 more days. Upon completion of 90 days of doxycycline therapy, the patient returned for further evaluation, reporting the cessation of transient swelling and the absence of pain. The total duration of antibiotic treatment comprised 90 days of doxycycline 100mg BID. Two months after discontinuation of antibiotics, the patient denied pain, swelling or limitation of activities, nor was effusion noted on physical exam.

## Discussion

When a patient presents with atraumatic knee effusion, especially in areas where Lyme disease is prevalent, Lyme arthritis should be investigated. The same can be said regarding septic arthritis in those with TKA. The waters begin to muddy when these two cross paths, which seems to be a rare event, although likely underreported. Because of the relatively few cases reported, Lyme PJI may evade the differential diagnosis of providers. The workup to determine the cause of joint effusion, including ESR, CRP, and synovial fluid cell count with differential, culture, gram stain, glucose analysis, and crystal analysis, is well understood by most physicians. But, given the prevalence of Lyme arthritis in areas that are endemic to Lyme disease, it may be of benefit to add a synovial fluid PCR test, the gold standard for diagnosis of Lyme arthritis.

While four of the five documented cases of Lyme PJI were treated with surgical intervention, two of these cases were diagnosed with Lyme PJI after irrigation and debridement had already been completed [6]. In the first case ever documented, the patient was treated with one week of oral doxycycline, then transitioned to 2 grams of ceftriaxone IV daily for six weeks with complete resolution of his symptoms [5]. Given our success with oral antibiotics, medical management may be a viable treatment for patients with acute, uncomplicated Lyme PJI. With *B. burgdorferi*’s ability to form biofilms, it is unknown whether nonsurgical intervention would be successful in patients with prolonged Lyme PJI [9,10]. Limitations of this study include the lack of long-term follow-up.

## Conclusions

While Lyme PJI is rare, it should be included in the differential of providers upon presentation of joint effusion. This is especially true in areas of high prevalence of Lyme disease, which includes the Northeastern United States. Medical management of Lyme PJI may be a viable option for patients who present with new onset symptoms, avoiding the risk of operative interventions. However, long-term follow-up is necessary to ensure there is no reoccurrence of symptoms. The lasting effects of Lyme PJI on the joint are unknown and should be examined further. The efficacy in more chronic presentations is unknown, and further investigation is needed.
